# Identifying habitat modification by Chinese pangolin in subtropical forests of southern China

**DOI:** 10.1111/1749-4877.12862

**Published:** 2024-07-23

**Authors:** Song SUN, Shichao WEI, Hongliang DOU, Shaolian CHEN, Haiyang GAO, Jinzhen YANG, Jingxin WANG, Yulin ZHANG, Yihang ZHANG, Ruiping GUO, Sheng ZHANG, Yumei DU, Ruiqi GAO, Yuanwen KUANG, Yan HUA

**Affiliations:** ^1^ Guangdong Provincial Key Laboratory of Silviculture, Protection and Utilization Guangdong Academy of Forestry Guangzhou China; ^2^ Guangdong Provincial Key Laboratory of Applied Botany and Key Laboratory of Vegetation Restoration and Management of Degraded Ecosystems South China Botanical Garden, Chinese Academy of Sciences Guangzhou China; ^3^ University of Chinese Academy of Sciences Beijing China; ^4^ College of Wildlife and Natural Protected Area Northeast Forestry University Harbin China; ^5^ College of Forestry and Landscape Architecture South China Agricultural University Guangzhou China

**Keywords:** burrow volume, habitat heterogeneity, *Manis pentadactyla*, mound coverage area, soil property, soil turnover

## Abstract

The excavation of Chinese pangolin (*Manis pentadactyla*) is expected to alter habitat heterogeneity and thus affect the functioning and structure of forest ecosystems. In this study, the bioturbation of Chinese pangolin on forest soils in three regions (Heping, Tianjingshan, and Wuqinzhang) across Guangdong province was quantified. Overall, a mean of 2.66 m^3^·ha^−1^ and 83.1 m^2^·ha^−1^ of burrows and bare mounds, respectively, was excavated by Chinese pangolin; the disturbed soils had significantly lower water content and P, C, available N concentrations, but higher bulk density, pH, and microbial abundance than those undisturbed soils. The unevenness of habitat heterogeneity improvement was mainly ascribed to the stronger soil disturbance caused in resting burrows by pangolins. Patterns of altering habitat heterogeneity were site‐specific, with high‐intensity soil disturbance occurring most in shrubs, meadows, steep habitats at high elevations, and mountain tops in Heping, while in broad‐leaved, coniferous and mixed coniferous and broad‐leaved forests away from human settlements in Tianjingshan and upper mountains at high elevations far away from roads and human settlements in Wuqinzhang. Road networks are the main interference for the burrow distribution in Heping and Wuqinzhang and should be programmed.

## Graphical abstract

In this study, we identified the critical role of Chinese pangolin in altering habitat heterogeneity, by quantifying habitat subsurface space and surface bare patches increased by pangolin burrows. We thus suggested that pangolins may profoundly affect vegetation renewal and animal distribution in forest ecosystems. Measurements like acceleration rejuvenation and conservation reintroduction of the pangolins may cascade to the recovery of habitat quality and even communities.

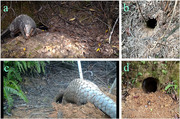

## INTRODUCTION

The destruction of primary forests worldwide leads to habitat fragmentation and subsistence resources loss, cascading to species extinction (Betts *et al.* 2019; Hansen *et al.* 2020). Restoration of forest vegetation has been quite successful in China (Tian *et al.* 2022); however, the declining habitat heterogeneity in secondary and plantation forests is equally worrisome (Gong & Tang 2016). Habitat heterogeneity affects microhabitat abundance and plays vital roles in biodiversity maintenance and faunal community resilience (Davidson *et al.* 2008; Bravo *et al.* 2009; Root‐Bernstein & Ebensperger 2012; Suggitt *et al.* 2018).

“Ecosystem engineers” are animal species that alter habitats through physical modifications (Jones *et al*. 1994, 1996). The most common “ecosystem engineers” are burrowing animals (Coggan *et al.* 2018), which can alter habitat heterogeneity by creating underground space and bare soil mounds (Crame & Willig 2005; Báldi 2008; Gooday *et al.* 2010). The burrowed microhabitats have considerable and semi‐permanent effects on micro‐topography, nutrient availability, and distribution (Davies *et al.* 2019), which provides multifunctional shelter for other organisms to hide, forage, and breed and for thermal buffering (Bragg *et al.* 2005; Desbiez & Kluyber 2013; Sun 2022).

The capacity of “ecosystem engineers” to improve habitat heterogeneity is usually identified by quantifying the volume of burrows. For example, European badgers (*Meles meles*) can dig up 12 m^3^ of burrows and excavate approximately 0.03 m^3^·ha^−1^·year^−1^ of soils (Coombes & Viles 2015). European bee‐eaters (*Merops apiaster*) can move approximately 0.00871 m^3^ of sand during nest construction (Casas‐Criville 2005). Generally, fluid displacement, burrow mold immersion, geometric computation based on morphometric measurements, and mathematical modeling of the burrow shape were used to estimate burrow volume (Kinlaw 1999; Smallwood & Morrison 1999; Whitford & Kay 1999; Bancroft *et al.* 2004; Bragg *et al.* 2005; Sawyer *et al.* 2012; Coombes & Viles 2015; Haussmann 2016). Burrow molds provide more accurate volume and morphometric measurements, which are particularly useful for complex or interconnected burrow systems (Reynolds & Wakkinen 1985). Geometric computations based on morphometric measurements are effective for single or multiple burrows (Eriksson & Eldridge 2014; Coombes & Viles 2015). However, studies assessing the landscape heterogeneity improvement of burrowing engineers are still scarce (Coombes & Viles 2015; Haussmann *et al.* 2016), although they provide scientific standards for the ecological conservation of these species (Coggan *et al.* 2018; Davies *et al.* 2019).

Underground soil piled up mounds have different properties from the surrounding surface soils, such as soil pH, concentration of carbon (C), nitrogen (N), phosphorus (P), and organic matter (Fleming *et al.* 2013; Mallen‐Cooper *et al.* 2019; Tania *et al.* 2020). Bare mounds can increase soil nutrient heterogeneity and have cascading effects on plant succession and animal utilization (Coggan *et al.* 2018), for example, altering the seed bank, promoting colonization by pioneer species, plant germination, and growth (Whitford & Kay 1999; Bancroft *et al.* 2005). At the same time, bare mounds provide shelter and thermal refugia for a range of commensal taxa (Warren & Büttner 2008; Fleischer *et al.* 2013). For example, the mounds created by European mole (*Talpa europaea*) are oviposition habitats for small copper (*Lycaena phlaeas*) within central European mesotrophic grasslands (Streitberger *et al.* 2014). To date, the ecological function and the morphological measurements of burrow mounds are rarely addressed.

Soil disturbances caused by burrowing animals are usually uneven due to the differences in burrow soil turnover mass and burrow distribution (Wu *et al.* 2004; Desbiez & Kluyber 2013; Halstead *et al.* 2020). It is essential to quantify not only the total capacity of soil turnovers but also their distribution patterns to evaluate the habitat heterogeneity improvement by burrowing animals (Jouquet *et al.* 2006; Di Blanco *et al.* 2020). Meanwhile, the fine‐scale distribution of soil disturbance is the basis for developing operational protection plans (Sharma *et al.* 2020; Wang *et al.* 2021; Zhang *et al.* 2021), which is a gap in ecosystem engineering biology.

The population of Chinese pangolin (*Manis pentadactyla*) has declined dramatically by up to 90% due to overhunting and habitat fragmentation within the past 40 years (Wu *et al.* 2002; Yin *et al.* 2016; Hua *et al.* 2020). Chinese pangolin is now critically endangered in China and listed as a threatened species by the IUCN (Jiang *et al.* 2016; Challender *et al.* 2019) and as a National First‐Class Protected Animal by China (National Forestry and Grassland Administration 2020). They were once widely distributed and are best known as termite predators (Shi 1985; Heath 1992) as well as skilled excavators (Lin 2011). A density of 110.8 burrows·ha^−1^ was reported in forests of Taiwan province (Lin 2011), implying that pangolin burrows are a key component of habitat heterogeneity within the Chinese pangolin range. However, the effects of pangolins on habitat heterogeneity improvement have been less addressed (Sun *et al.* 2021); despite that, the properties of pangolin burrows, such as diameter, depth, and density, were frequently documented (Wu *et al.* 2004; Fan 2005; Lin 2011). The lasting decline in the pangolin population inevitably reduces the total mass of soil turnover, which may considerably affect habitat heterogeneity and biodiversity in forests (Connell 1978; Root‐Bernstein & Ebensperger 2012).

During a multi‐year field investigation (2019–2023), we found that the remnant Chinese pangolin populations in the Guangdong province of southern China are mainly distributed in secondary and plantation forests. In this study, we selected three regions across Guangdong province (forests in Heping, Tianjingshan, and Wuqinzhang) where Chinese pangolins are frequently founded (Sun 2022), collected morphological data from 299 burrows, and calculated their soil turnover capacity at the landscape scale according to the standard procedure (Garkaklis *et al.* 2004; Casas‐Criville 2005; Valentine *et al.* 2013). We sampled soils from 103 fresh burrows in the above three regions to analyze the soil property alteration and assess the soil disturbance distribution pattern by pangolin (Kerley *et al.* 2004; Van Vuren & Ordeñana 2012; Wang *et al.* 2021). Results are expected to provide a critical understanding of the ecological roles of Chinese pangolin in proving habitat heterogeneity and restoring habitat quality.

## MATERIALS AND METHODS

### Study site

**Figure 1:**
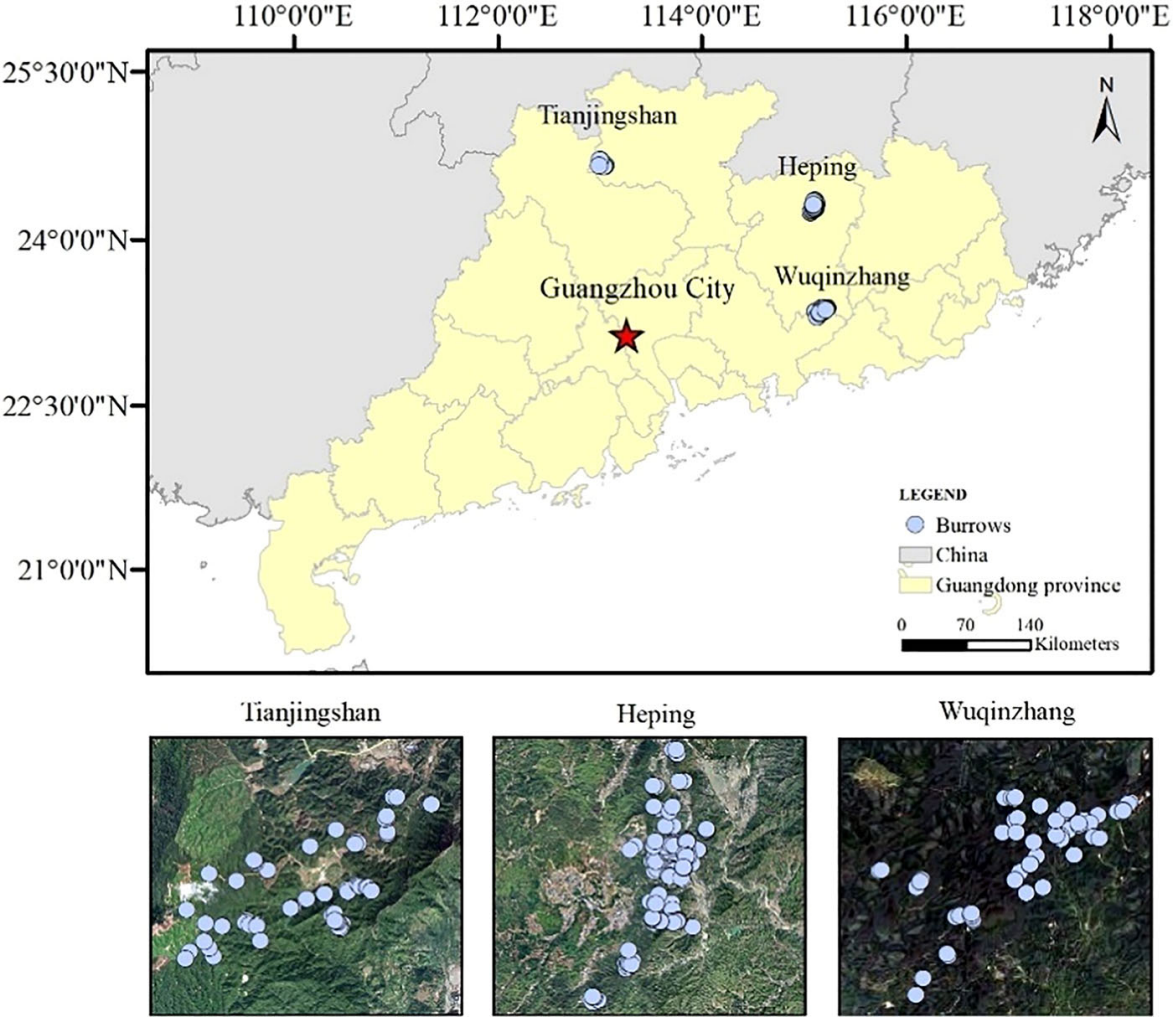
Burrow distribution of Chinese pangolins in forests of Tianjingshan, Heping, and Wuqinzhang across Guangdong province.

The investigation was conducted in forests of three regions across Guangdong province, southern China: (1) Wuqinzhang in Huizhou (22.5442°–23.3881°N; 114.5528°–115.4203°S) in December 2020; (2) Heping in Heyuan (24.0833°–24.7°N; 114.6833°–115.2667°S) from January to April 2021; and (3) Tianjingshan in Shaoguan (24.5333°–24.7666°N; 112.5°–113.25°S) from September to November 2020 (Fig. 1). Wuqinzhang is located in southern Guangdong and has a mean annual temperature (MAT) and mean annual precipitation (MAP) of 20°C and 1950 mm, respectively (Li & Wu 1999). Heping is located in east‐central Guangdong and has a MAT and MAP of 19.7°C and 1717.1 mm, respectively (Huang *et al.* 2019). Tianjingshan is located in northern Guangdong and has a MAT and MAP of 17.7°C and 1705 mm, respectively (Li *et al.* 2020).

### Field investigation

Pangolin burrows were investigated by survey transect covering a distance of 3–5 km. A total of 142 transects (56, 62, and 24 in Heping, Tianjingshan, and Wuqinzhang, respectively) covering a total distance of 685 km were investigated (see Supporting Information 1). Pangolin burrows were searched along each transect, identified according to the specific distinguish methods described in Supporting Information 2 (Lin 2011; Zhang *et al.* 2021), and in the Heping forest, some burrows were located in burnt areas.

### Morphometric measurement and burrow calculation

Mound coverage area was determined by measuring the length of the vertical (*A*) and horizontal axes (*B*) parallel to the ground according to the equation: *S* (mound) = π × *A*/2 × *B*/2 (Borchard & Eldridge 2011; Coombes & Viles 2015).

**Figure 2:**
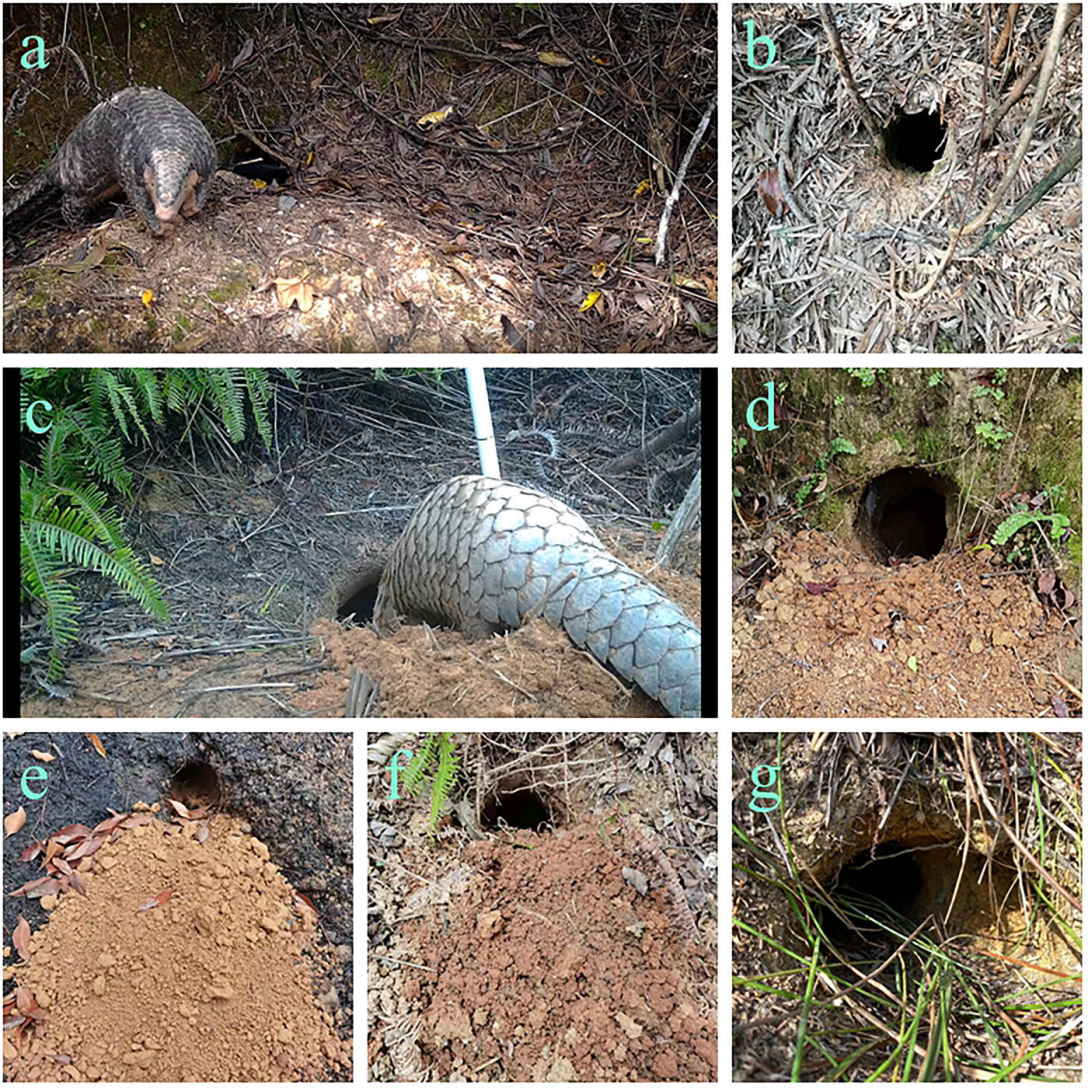
A close‐up of a pangolin burrow, where subterranean soil has been excavated into a mound outside the entrance. Transverse diameter and shaft diameter of these burrows are: (a) 16 and 14 cm; (b) 13 and 12 cm; (c) 17 and 15 cm; (d) 16 and 16 cm; (e) 14 and 11 cm; (f) 14 and 11 cm; (g) 18 and 16 cm. Note: Some of the burrows, such as (e) are located in the burned area.

Pangolin burrows usually have a simple structure and regular shape, with oval or round tunnel sections and thus can be regarded as tortuous elliptical columns (Fig. 2). The long (*L*) and short diameters (*S*) of the tunnels at the burrow entrance were measured with a long‐arm vernier caliper (Zimmerman 1990; McDonough *et al.* 2000). Burrow depth (*D*) was detected by inserting a flexible rod into the burrow bottom (Doonan & Stout 1994; McDonough *et al.* 2000). Burrow volume (*V*) was determined by the equation: *V* (burrow) = π × *L*/2 × *S*/2 × *D* (Sawyer *et al.* 2012; Eriksson & Eldridge 2014).

To maximize the function of healthy/sub‐healthy pangolin populations to improve habitat heterogeneity at the landscape scale, we used burrow density data (*D*b) recommended by Lin (2011) to determine the total burrow volume (TV) and total mound coverage area (TS) per ha as follows according to Garkaklis *et al.* (2004), Casas‐Criville (2005), and Valentine *et al.* (2013):

$$\mathrm{TV}=\sum_{i=1}^{V\left(\mathrm{burrow}\right)}/i\times Db.$$

$$\mathrm{TS}=\sum_{i=1}^{S\left(\mathrm{mound}\right)}/i\times Db.$$

where *V*_(burrow)_ is the volume of the pangolin burrow, *S*_(mound)_ is the mound coverage area, *D*b is the burrow density, and “*i*” is the serial number of burrows.

### Soil sampling and measurement

Fresh burrows were selected for soil sampling to eliminate the impact on soil properties from environmental factors (sunlight, rainfall etc.; for burrow age identification methods, see Supporting Information 3). Four samples were collected from each burrow: one from the center of the burrow mound (the intersection of the horizontal and vertical axes of a burrow mound) and three from 5 m away from the center where there was no burrow disturbance (control soil) (Gharajehdaghipour *et al.* 2016). Prior to sampling, mound surface litter and consolidated soil were scraped. A total of 412 soil samples (103 × 4) from 103 pangolin burrows were collected (61 in Heping, 22 in Tianjingshan, and 20 in Wuqinzhang; Table 2). The soils were air‐dried and ground for detecting soil bulk density; water content; soil pH; total C, N, and P and available N [NH_4_^+^, NO_3_^−^]; and microbial biomass C, N, and P concentrations. All analyses were performed according to the standard of Liu *et al.* (1996).

### Burrow microhabitat survey

When the distance between two burrows was less than 20 m, only the first burrow was selected to record microhabitat information (to reduce false duplications of microhabitat data and retain proximity distribution characteristics of pangolin burrows). Pangolin burrows were grouped into foraging and resting burrows (see Supporting Information 4; Wu *et al.* 2003, 2004; Fan 2005; Dorji 2017). Variables that may affect soil turnover and burrow categories (Bc) (Wu *et al.* 2003; Bhandari & Chalise 2014; Dorji 2017; Sharma *et al.* 2020) were recorded or extracted. Environmental variables such as elevation, slope, slope direction, slope position, forest type, and soil type and the distance to the nearest river were recorded. Anthropogenic factors were the distance to the nearest human settlement and the distance to the nearest foot trail (version 10.8, Wu *et al.* 2003; Katuwal *et al.* 2017; Dnpwc & Dof 2018; Sharma *et al.* 2020). Details of the variables are listed in Table 1.

**Table 1:** The classification of variables

Variables	1	2	3	4	5	6	Reference
Burrow category	Foraging burrow	Resting burrow					Karawita *et al.* (2018); Sun *et al.* (2021)
Slope position	Lower	Middle lower	Middle	Middle upper	Upper	Top	Wu *et al.* (2003); Dnpwc and Dof (2018)
Soil type	Yellow soil	Red soil	Black soil	Yellow sandy soil	Red sandy soil	Yellow clay	Sharma *et al.* (2020)
Forest type	Broad‐leaved forest	Coniferous forest	Mixed needle and broad‐leaved forest	Shrub wood	Grassy meadows		Wu *et al.* (2003, 2004)

### Data analysis

**Figure 3:**
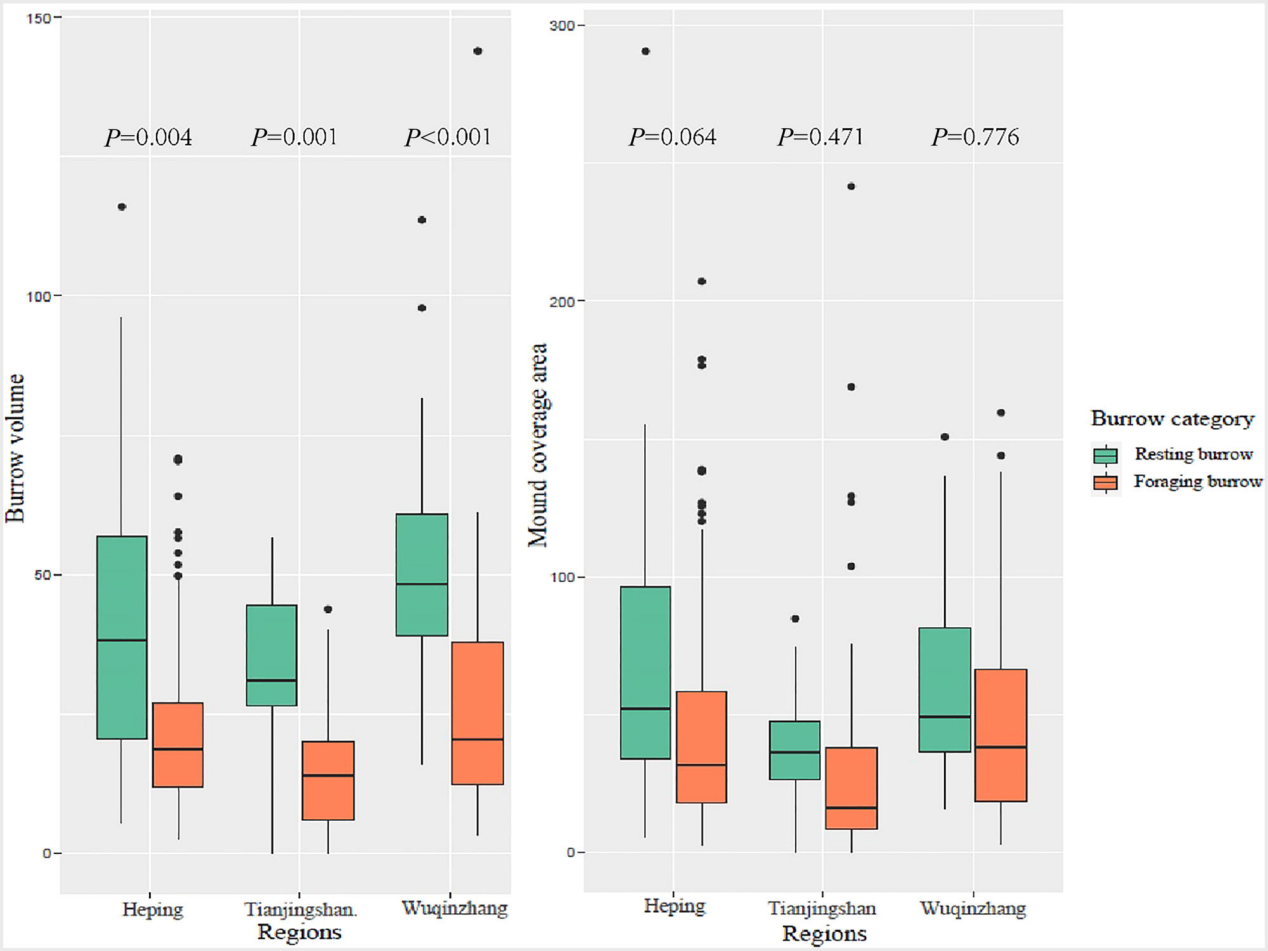
The difference of burrow volume and mound coverage area between resting burrows and foraging burrows in three regions.

**Figure 4:**
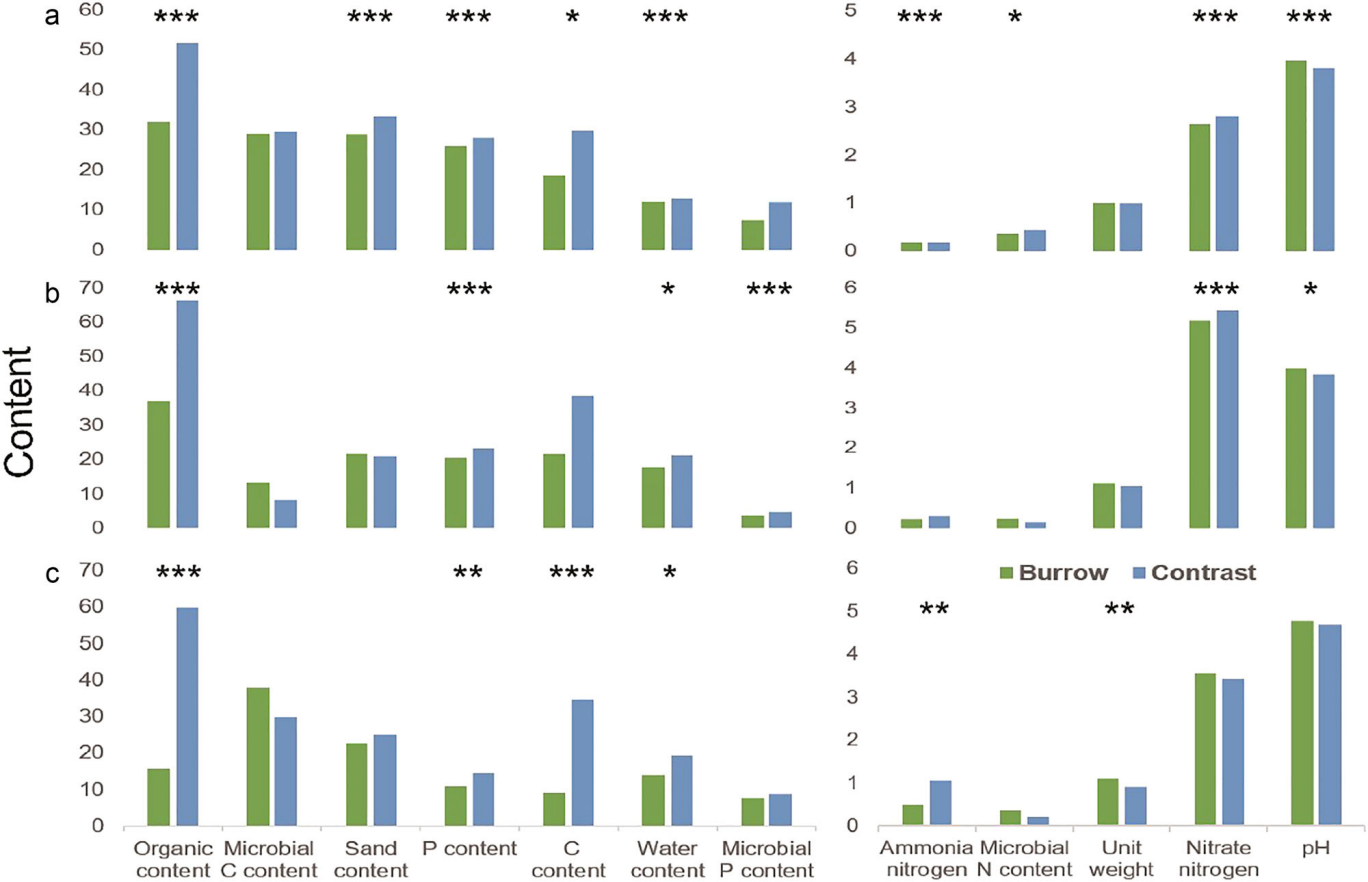
The difference of soil properties between pangolin burrows and control sites (natural habitat) in three regions. A, B, and C refer to Heping, Tianjingshan, and Wuqinzhang regions, respectively. Significant codes: 0.0001 “^***^,” 0.001 “^**^,” 0.01 “^*^,” 0.05 “.,” 0.1 “,” 1; of these indexes, P content has been divided by 10 for visualization purposes.

The morphometric data of Chinese pangolin burrows in this study are shown in Table 2. The differences in burrow volume and mound coverage area between resting and foraging burrows were compared (Fig. 3). Because resting burrows are generally deeper than foraging burrows (Wu *et al.* 2003, 2004; Fan 2005; Dorji 2017), we treated burrow category not only as a covariate affecting soil turnover but also as a response variable for habitat variables in this study. The differences in soil properties (Table S1, Supporting Information) between burrow soils and control soils were compared (Fig. 4).

**Table 2:** Burrow size, burrow volume, and mound coverage area

Location	Minimum	Maximum	Mean	Estimated SD
Long diameter (cm)	8	23	14.54	2.26
Short diameter (cm)	7	23	12.79	2.38
Horizontal axis (cm)	30	150	68.87	19.54
Vertical axis (cm)	47	258	123.97	40.55
Depth (cm)	20	560	153.22	99.03
*V*_(burrow)_ (m^3^)	0.0025	0.116	0.024	0.017
*S*_(mound)_ (m^2^)	0.13	2.98	0.75	0.46
*V*_(resting burrow)_ (m^3^)	0.0053	0.116	0.044	0.0267
*V*_(foraging burrow)_ (m^3^)	0.0025	0.024	0.021	0.0159
*S*_(resting burrow)_ (m^2^)	0.271	2.98	0.813	0.055
*S*_(foraging burrow)_ (m^2^)	0.13	1.16	0.667	0.008
TV (m^3^·ha^−1^)	2.66 ± 1.88			
TS (m^2^·ha^−1^)	83.1 ± 51.0			

**Figure 5:**
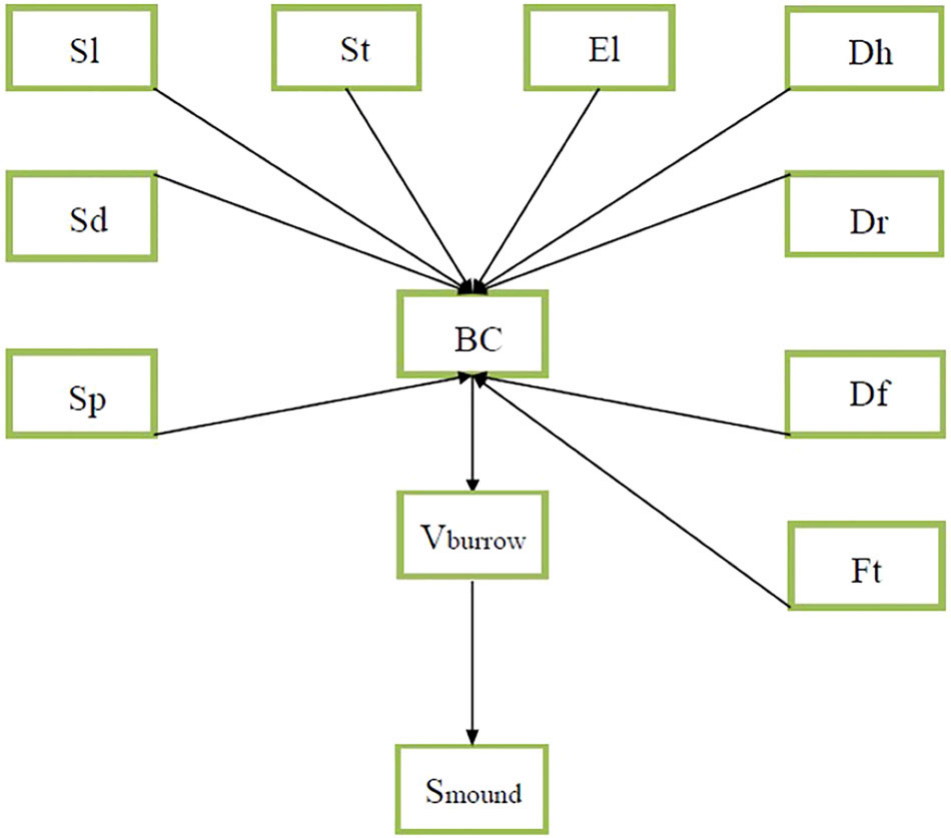
Conceptual model of hypothesized relationships showing the direct and indirect effects of habitat and anthropogenic variables on burrow volume and mound coverage area. The diagram should be interpreted as a cascade of effects. The hypothesized cascade is that habitat and anthropogenic variables, elevation (El), slope (Sl), slope direction (Sd), slope position (Sp), soil type (St), forest type (Ft), distance to nearest foot trail (Df), distance to nearest human settlement (Dh), and distance to nearest river (Dr), directly affect burrow category (Bc) and then these nine variables indirectly affect burrow volume (*V*_burrow_) and mound coverage area (*S*_mound_).

A structural equation model (SEM) was adapted to reveal the soil turnover distribution pattern by evaluating potential connections between habitat variables and burrow category, burrow volume, and mound coverage area (Fig. 5, Note: The blurred edges of the mounds in many of the burrows in the Tianjingshan region made it impossible to accurately calculate the mound coverage area, so this variable was not included in the model). Linkages between the burrow category and elevation, slope, slope position, slope direction, soil type, forest type, distance to the nearest river, distance to the nearest foot trail, and the distance to the nearest human settlement were determined as shown in Fig. 5. The goodness‐of‐fit for the model was determined from the maximum likelihood chi‐square tests. If *P*‐value was greater than 0.05, the model fits produced covariance matrices that were not significantly different from the observed covariance matrices (Kumar *et al.* 2017). Considering that the chi‐square test is influenced by sample size and data distribution, we reported the comparative fit index (CFI), root‐mean‐square error of approximation (RMSEA), and standardized root mean square residuals (SRMR). A good model fit was indicated by CFI > 0.95, RMSEA < 0.05, and SRMR < 0.08 (Rosseel 2012). We utilized Akaike's (AIC, Akaike 1974) and Bayesian information criterion (BIC, Hossain & King 1997) to compare the models with significant fits that determine the model closest to the unknown process that generates the patterns represented by the data (Burnham & Anderson 2002). To validate the specification of the SEM, the bivariate relationships representing the directional causal path in Fig. 5 were assessed using partial regression analysis. All analyses were performed using the “lavaan” package (Rosseel 2012) in R (version 4.1.3).

## RESULTS

A total of 299 Chinese pangolin burrows (185 in Heping, 60 in Tianjingshan, and 54 in Wuqinzhang) including 259 foraging burrows and 40 resting burrows, were identified in this investigation. The total burrow volume and the total mound coverage were 2.66 ± 1.88 m^3^·ha^−1^ (approximately 2.76 ± 1.95 t·ha^−1^) and 83.1 ± 51.0 m^2^·ha^−1^, respectively (Table 2), with a mean value of 0.024 m^3^ and 0.75 m^2^, respectively. The total mass of soil turnover and mound coverage area varied significantly across the burrows (*P* < 0.05). The resting burrows had significantly larger volumes than the foraging ones while both the two types of burrows had similar bare mounds (Fig. 3).

In Heping, there were significant differences in soil properties between the burrow and control soils, with higher available N, pH, and bulk density in the burrow than in the control soil. In Tianjingshan, burrow soils had higher sand content, bulk density, pH, and microbial biomass C and N concentrations than control soils. In Wuqinzhang, burrow soils had higher bulk density, pH, nitrate nitrogen, and microbial biomass C and N (Fig. 4; Table S1, Supporting Information).

**Figure 6:**
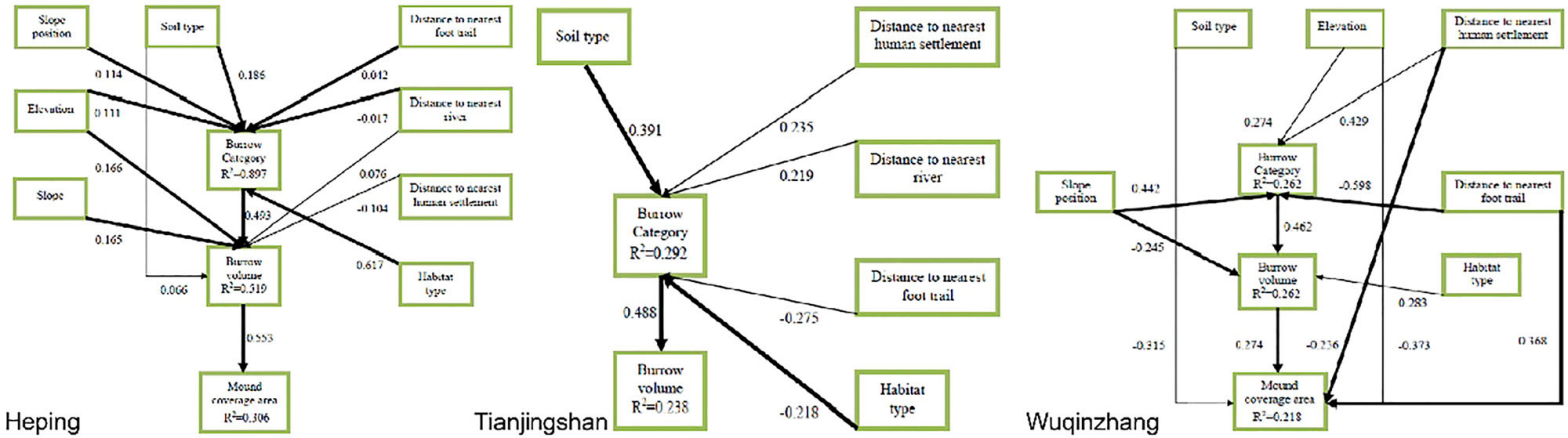
Structural equation models (SEMs) of three regions. In the final SEM of the Heping, linking distance to nearest foot trail, distance to nearest river, slope position, elevation, soil type, and forest type to burrow category; linking burrow category, distance to nearest human settlement, distance to nearest river, slope, elevation, and soil type to burrow volume; and linking burrow volume to mound coverage area. In the final SEM of the Tianjingshan, linking distance to nearest human settlement, distance to nearest foot trail, distance to nearest river, soil type, and forest type to burrow category; linking burrow category to burrow volume. In the final SEM of the Wuqinzhang, linking distance to nearest human settlement, distance to nearest foot trail, elevation, and slope position to burrow category; linking burrow category, slope position, forest and type to burrow volume; and linking distance to nearest human settlement, distance to nearest foot trail, elevation, and soil type to mound coverage area. Bold lines represent statistically significant paths (*P* ≤ 0.05). The path coefficients between the variables are plotted on the path. The arrow indicates the direction of regression, and the coefficients are standardized for each causal path.

In Heping, elevation and slope had significant effects on burrow volume (Fig. 6). Neither the slope direction, slope, nor the distance to the nearest human settlement had significant effects on the burrow category, while elevation, soil type, and distance to the nearest river had direct effects on burrow category and burrow volume. Forest type had the strongest effects on the burrow category (*r* = 0.617). Furthermore, the burrow category had positive and direct effects on burrow volume (*r* = 0.493), and burrow volume had positive effects on the mound coverage area (*r* = 0.553). The direct and indirect standardized path coefficients of the two variables are exhibited in Table 3. The resultant SEM models (χ^2^ = 2.510, df = 14.000, *P* = 1.000, CFI = 1.000, SRMR = 0.124, RMSEA = 0.000) had good fit (Fig. 6).

**Table 3:** The path coefficient between variables

Variables	Dh	Df	Dr	Sl	Sd	Sp	Ft	El	St	BC	*V*_burrow_
Heping Region											
BC		−0.042	−0.017			0.114	0.617	0.111	0.186		
*V*_burrow_	−0.104	−0.021	−0.068	0.165		0.056	0.304	0.221	0.158	0.493	
*S*_mound_	−0.058	−0.011	−0.038	0.091		0.031	0.168	0.122	0.087	0.273	0.553
Tianjingshan Region											
BC	0.235	−0.275	0.219				−0.218		0.391		
*V*_burrow_	0.115	−0.134	0.107				−0.106		0.191	0.488	
Wuqinzhang Region											
BC	0.429	−0.598				0.442		0.274			
*V*_burrow_	0.198	−0.276				−0.041	0.283	0.127		0.462	
*S*_mound_	−0.182	0.092				−0.011	0.078	−0.338	−0.315	0.127	0.274

Abbreviations of covariates in the table indicate the full names are as follows: El, elevation; Sl, slope; Sd, slope direction; Sp, slope position; St, soil type; Ft, forest type; Df, distance to nearest foot trail; Dh, distance to nearest human settlement; Dr, distance to nearest river; Bc, burrow category; *V*_burrow_, burrow volume; *S*_mound_, mound coverage area.

In Tianjingshan, slope, slope direction, and slope position had no significant effects on burrow category or volume. Soil type (*r* = 0.391), distance to the nearest human settlement (*r* = 0.235), and distance to the nearest river (*r* = 0.219) showed positive, but the distance to the nearest foot trail (*r* = −0.275) and forest type (*r* = −0.218) showed negative effects on the burrow category. The burrow category had the strongest effects on burrow volume (*r* = 0.488). The resultant SEM models (χ^2^ = 4.244, df = 4.000, *P* = 0.374, CFI = 0.992, SRMR = 0.038, RMSEA = 0.032) had a good fit to the data for the purpose variables (Fig. 6).

In Wuqinzhang, slope position and forest type showed direct effects on burrow volume; soil type, distance to the nearest human settlement, and distance to the nearest foot trail had significant and direct effects on mound coverage area. Slope direction, slope, and distance to the nearest river had no significant effects on the burrow category, burrow volume, or mound coverage area. Furthermore, the distance to the nearest foot trail had negative effects on the mound coverage area via burrow category and burrow volume (*r* = −0.076). Elevation and distance to the nearest human settlement showed indirect effects via burrow category and burrow volume on the mound coverage area (see Table 3). Slope position had direct negative effects (*r* = −0.245) and positive effects via the burrow category on burrow volume (*r* = 0.204). Soil type had negative direct effects on the mound coverage area (*r* = −0.315). The forest type had positive direct effects on burrow volume (*r* = 0.283). The resultant SEM models (χ^2^ = 10.279, df = 9.000, *P* = 0.328, CFI = 0.989, SRMR = 0.027, RMSEA = 0.051) had a good fit with the data for the purpose variables (Fig. 6).

## DISCUSSION

### Vertical habitat heterogeneity improvement

Pangolins occupy key niches in ecosystems through soil disturbance, rather than just termite predators as traditionally assumed (Wu *et al.* 2005; Thapa 2013). In this study, we assessed the habitat heterogeneity alteration caused by pangolins for the first time by quantifying the capacity of soil turnover at the landscape scale. The average volume of pangolin burrows (0.024 m^3^) is relatively small than those of similar‐sized burrowing animals, such as *Proteles cristatus* (0.672 m^3^, Richardson 1985), *Otocyon megalotis* (0.251 m^3^, Skinner & Smithers 1990), *Dasypus novemcinctus* (0.0345 m^3^, Sawyer *et al.* 2012), and *M. meles* (12 m^3^, Coombers & Viles 2015). However, the total soil turnover mass at the landscape scale (2.66 ± 1.88 m^3^·ha^−1^) is larger than *M. meles* (0.03 m^3^, Coombers & Viles 2015), which is likely due to the strong burrowing ability of pangolins (Lin 2011; Sun *et al.* 2021) and to the fact that pangolins hardly reuse old burrows (Wu *et al.* 2004).

We found that burrow digging of pangolins profoundly expanded the underground space thus significantly increasing the habitat vertical heterogeneity, coupled with the multiple resources provided by the burrows' cascading effects on biological community distribution. The results identified the considerable influences on processes and functions and performance of service of pangolins in forest ecosystems. For instance, more than 65 animal species can use pangolin burrows, which provide thermal buffering for at least 40 species (Sun 2022) and food resources (e.g., termites, ants, seeds, small vertebrate prey) and shelter from predators for commensal species (Bragg *et al.* 2005; Sawyer *et al.* 2012; Coombes & Viles 2015; Haussmann 2016; Dawson *et al.* 2019). At the same time, commensal species attracted and concentrated by burrow resource form a complexly structured food web due to their inter‐ and intraspecific relationships (predation, competition, etc.), which contributes to ecosystem stability and resistance (Krause *et al.* 2003; Thebault & Fontaine 2010; Dawson *et al.* 2019).

### Surficial habitat heterogeneity improvement

Our results showed the excavation by pangolins reduced soil water and sand content, increased soil bulk density, and partially explained the drying and surface compaction phenomenon (reduced permeability) of mounds. However, soil nutrients in the mounds decreased contrary to the results of studies in arid and semi‐arid regions (Bragg *et al.* 2005; Eldridge *et al.* 2012; Chapman 2013). This is manifested by a decrease in P, C, and available N concentrations, which may be due to the relatively dry surface of the mounds and the accumulation of less litter and humus (James *et al.* 2010). The disturbance increased the soil pH and microbial abundance, which was conducive to the availability of soil nutrients utilization by plants and the decomposition of organic and mineral matter (Shen *et al.* 2021; Serna‐Chavez *et al.* 2013). Bare mounds also improved surficial habitat heterogeneity, especially for habitats with sparse or monoculture vegetation. For example, the dense *Dicranopteris dichotoma* in the understory of southern China's forest often hinders shrub or tree regeneration (Yang *et al.* 2021). The excavation of pangolin burrows can destroy the rooting blanket layer of *D. dichotoma* and create bare conditions for the invasion and colonization of other species (Wu *et al.* 2004; Wang *et al.* 2021). Coupled with the soil properties of the mound, we suggest that the mounds may be conducive to the settlement of pioneer plant species that are drought‐resistant and have low nutrient requirements (e.g., *Triadica sebifera* (Linnaeus) Small). Thus, pangolin burrow microhabitats may facilitate early successional processes in habitats, which contradicts the notion that ecosystem engineers have a smaller impact in humid and semi‐humid regions (Davies *et al.* 2019).

### Unevenness of habitat heterogeneity improvement by pangolin

Differences in the mass of soil turnover among pangolin burrows determined the unevenness of habitat heterogeneity improvement. Three SEM results showed that soil turnovers in the forests by pangolins were mainly dependent on the burrow category, despite the fact that some environmental variables had direct or indirect effects on them (Table 3). Indicated by volume (0.044 m^3^ vs. 0.021 m^3^) and mound coverage area (0.813 m^2^ vs. 0.667 m^2^, Table 2), we found that soil disturbance in resting burrows is relatively larger than in foraging burrows. Thus, the unevenness of habitat heterogeneity improvement in pangolins is associated with the distribution of resting burrows.

In addition, patterns of habitat heterogeneity improvement in pangolins varied across the three regions, likely due to the different vegetation structures or human disturbance intensity (Suwal *et al.* 2020; Tamang *et al.* 2022). In Heping and Wuqinzhang, a bigger mass of burrow overturn soil was found in grassy meadows (*r* = 0.304 and *r* = 0.283), rather than pure forests (e.g. *Pinus* forests) despite their relatively large area. The grassy meadows were mostly distributed in the hill‐tops with high elevation (*r* = 0.221; *r* = 0.127) and slope position (*r* = 0.056; *r* = 0.041), which had low plant diversity due to the dense cover of *D. dichotoma* or *D. pedata*; the distribution of burrows creates colonized bare ground for other pioneer plant species. However, in Tianjingshan, pangolins overturned more soil in broad‐leaved and coniferous and broad‐leaved mixed forests (*r* = −0.218); the elevation and slope position thus had no significant effects on soil turnover (Fig. 6). Coniferous forests are basically plantation forests (*Cunninghamia lanceolata*) with a bare understory. Pangolin burrows significantly increased surficial heterogeneity and provided shelter and foraging resources for organisms. Pangolins preferred to overturn more soils in sandy loam and yellow clay in Heping and Tianjingshan but had no obvious preference in Wuqinzhang (Table 3), which is inconsistent with Bhandari and Chalise (2014) and Sharma *et al.* (2020) who found that pangolin burrows were mostly distributed in red and brown soils. The possible reason is that sandy loam is soft and easy to dig, especially after rainfall and clay contributes to more solid burrow structure and longer service life.

The distance to the nearest river always affects burrow habitat selection (Wu *et al.* 2003; Suwal *et al.* 2020; Waseem *et al.* 2020) but did not significantly affect soil disturbance by pangolins in this study. The model results showed that pangolins tend to overturn more soil in areas far from human settlements in Tianjingshan and Wuqinzhang. The results are consistent with previous studies (Wu *et al.* 2003; Bhandari & Chalise 2014; Karawita *et al.* 2018; Shrestha *et al.* 2021), which suggested that pangolins prefer to inhabit forested areas rather than human‐disturbed areas. However, we found that resting burrows were frequently close to roads in the Heping and Wuqinzhang, due to the dense road network passing through the core distribution of pangolin. The programing of road networks should be considered in future conservation efforts, since vehicles and pedestrians may affect pangolin activity and increase the probability of discovering burrows.

### Implication for pangolin conservation

In this study, we identified the ecological roles of Chinese pangolin in improving habitat heterogeneity and identified that pangolin can profoundly affect vegetation renewal and animal distribution in forest ecosystem. We suggest that the forest lands with dense resting burrows should be strictly protected as the core area pangolin conservation or reintroduction and that the road networks in this area should be carefully programmed to reduce human interference. Furthermore, burrow soil disturbance is more pronounced in areas of higher habitat homogeneity, for habitat heterogeneity improvement, such as meadows at high elevations, or on steep hillsides and mountains in Heping, in coniferous forests far away from human settlements in Tianjingshan, and in the upper mountains at high elevations in Wuqinzhang. Increased conservation efforts to rejuvenate wild populations and attempted reintroductions of pangolins in their historical range would benefit habitat quality and even biome recovery (Scheffers *et al.* 2014; McGowan *et al.* 2020), providing opportunities for studying the critical roles of pangolins in forest ecosystems.

## CONFLICT OF INTEREST STATEMENT

The authors declare that they have no known competing financial interests or personal relationships that could have appeared to influence the work reported in this paper.

## Supporting information


**Supporting Information 1** Survey transects of pangolin burrow investigation in three region
**Supporting Information 2** The difference between pangolin burrow and non‐pangolin burrows
Supporting Information 3
Supporting Information 4
**Table S1** Changes of soil properties
