# Supplementary material for: Identifying habitat modification by Chinese pangolin in subtropical forests of southern China
Source: Integr Zool. 2024 Jul 23;20(2):361–75. doi: 10.1111/1749-4877.12862 (PMC11897934; doi:10.1111/1749-4877.12862)
Supplement: Supplementary file 1 — Supporting Information 1 Survey transects of pangolin burrow investigation in three region Supporting Information 2 The difference between pangolin burrow and non‐pangolin burrows Supporting Information 3 Supporting Information 4 Table S1 Changes of soil properties [file INZ2-20-361-s001.pdf]

Supporting Information 1

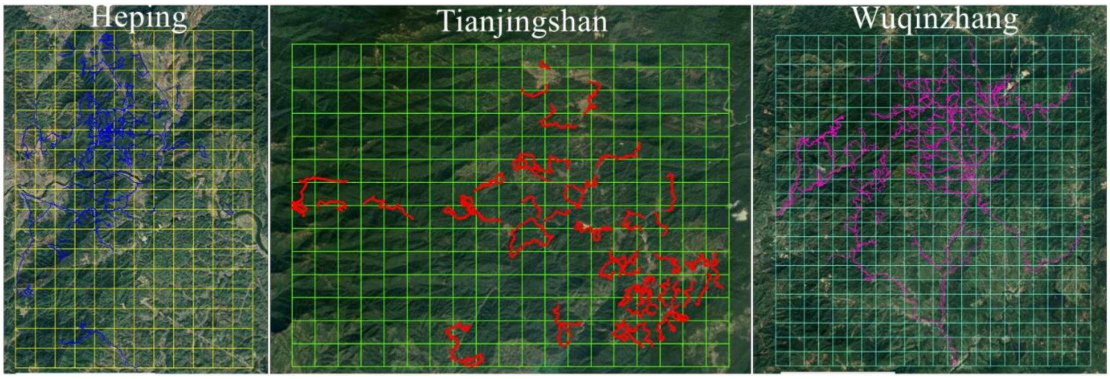

Survey transects of pangolin burrow investigation in three regions. The grid size in the figure is 1km x 1km, and these kilometer grids were used as the smallest unit for sampling during the survey. A total of 142 transects (56, 62, and 24 in Heping, Tianjingshan and Wuqinzhang, respectively) covering a total distance of 685 km was investigated.

Supporting Information 2

The difference between pangolin burrow and non-pangolin burrows

Mice burrows:

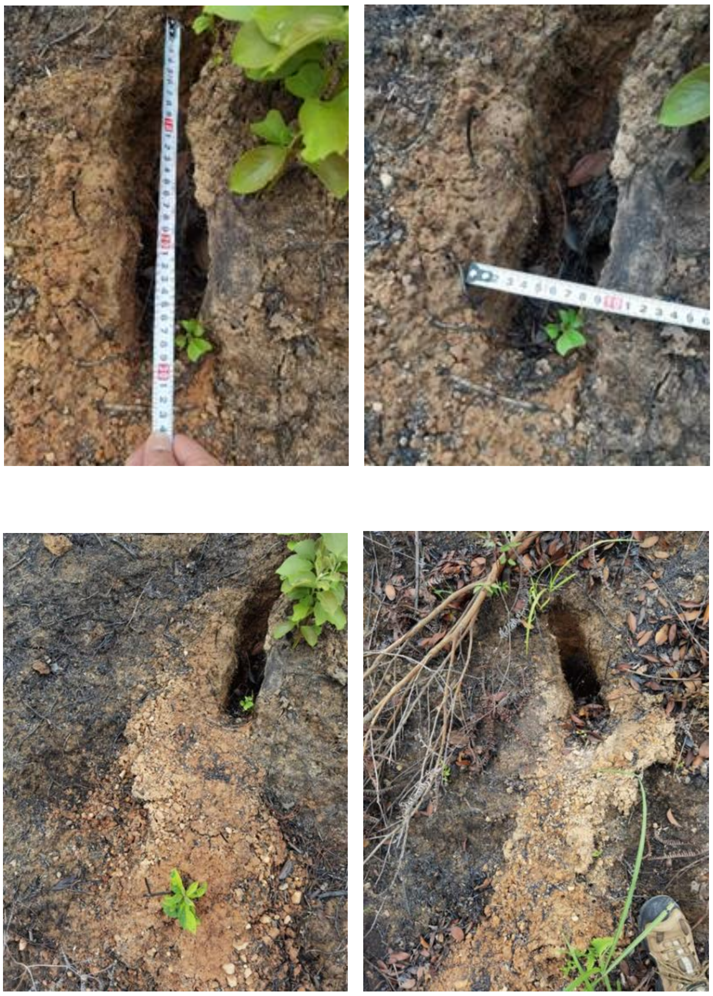

Distinguishment: most of them are burrows of Coxing's White-bellied Rats (*Niviventer coninga*). The shape of mice burrow entrance is quite different from that of pangolin burrow: the entrance of mice burrow is strip shape, and the long diameter (≥30cm) is much larger than the short diameter (≤10cm); the pangolin burrow entrance is round or ellipse.

Bamboo rat burrows:

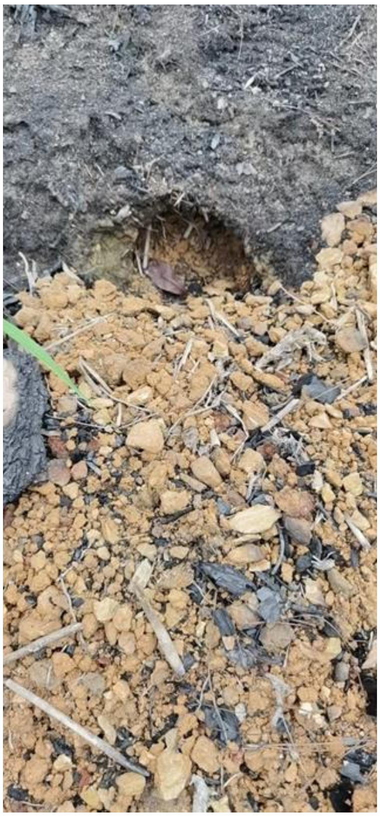 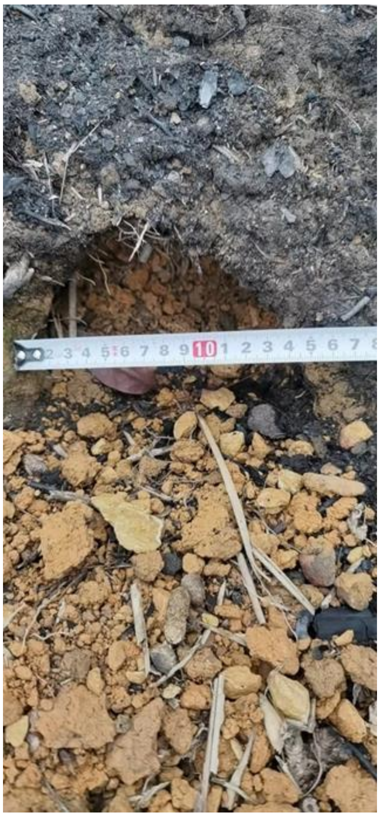

Characters: The shape and size of fresh bamboo rat burrows are similar to pangolin burrows, which can be easily confused. The differences are as follows: 1) There are filamentous traces left by hair rubbing against the burrow wall, which are different from the smooth plane caused by scale rubbing against the burrow wall; 2) Bamboo rats often bite off the roots at the burrow entrance, but the roots at the pangolin burrow entrance remain intact, because pangolin has no teeth; 3) There are usually grass stalks of similar length buried in bamboo rat burrows, which are left by bamboo rats after eating, and there are feces excreted by bamboo rats on the surface of the mounds outside the burrows; no pangolin burrow has this feature

Porcupine (*Hystrix brachyura hodgsoni*) burrows:

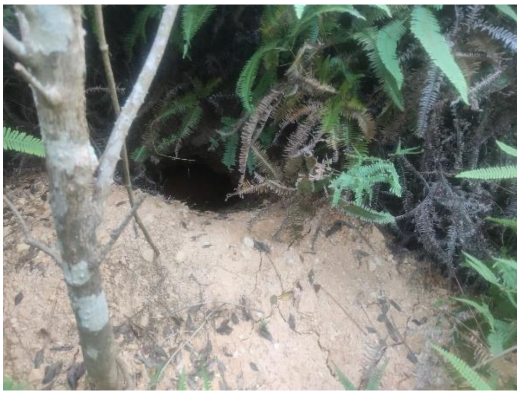 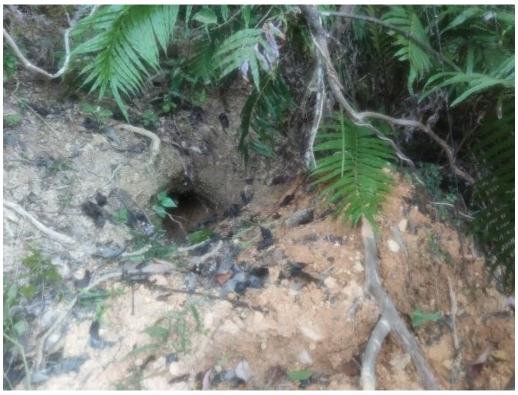

Characters: Porcupine burrows are relatively rare, and the biggest difference from pangolin burrows is that porcupine burrows are larger, usually with a diameter of more than 30cm, making them easier to distinguish.

Supporting Information 3

There are no public reports on the determination of age of pangolin burrows. Combining the observation experience of field guides (who are familiar with the animal's habits) and the comparison of a large number of burrows, we classify the age of the burrows into 4 categories:

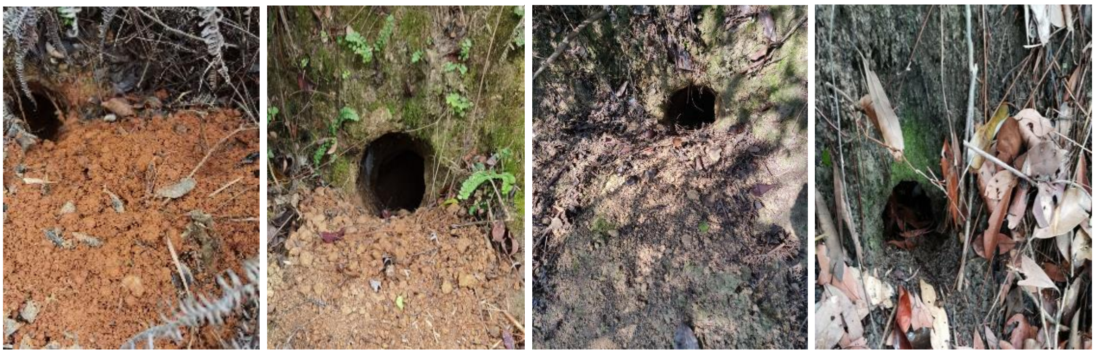

From left to right:

1. Fresh burrows within 1 month: burrows with intact entrance and no weed growth, the mounds soil are moist and with brightly color, easy to spot.
2. Fresh burrows within 1 year: burrows with intact entrance and smooth tunnel, the mounds are elliptical, with the color remaining distinctly different from the surrounding soil.
3. Burrows within 2 year other than 1 year: burrows with worn entrance and intact tunnel; the mound soil is close in surrounding soil, and there are trees and shrubs growing on the mound and in the burrow entrance.
4. Burrows older than 2 years: burrows with crumble entrance and collapse tunnel; the mounds are close to disappearing.

Supporting Information 4

Categories were determined based on i) the burrow use frequency (pangolin frequently utilizes the resting burrow, its burrow entrance is relatively smooth, and the upper edge of the entrance often contains friction marks by pangolin scales; in contrast, foraging burrow entrance appears rough), and ii) marks of repeated excavations in the burrow (pangolins remodel their resting burrows, but do not dig foraging burrows twice).

Resting burrows:

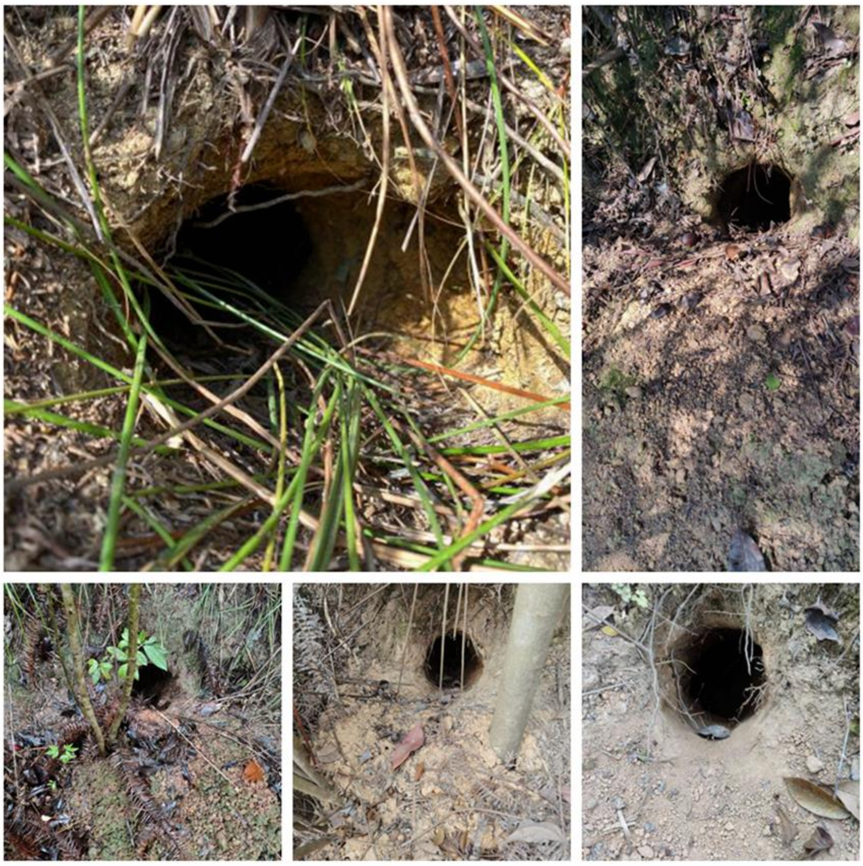

Foraging burrows:

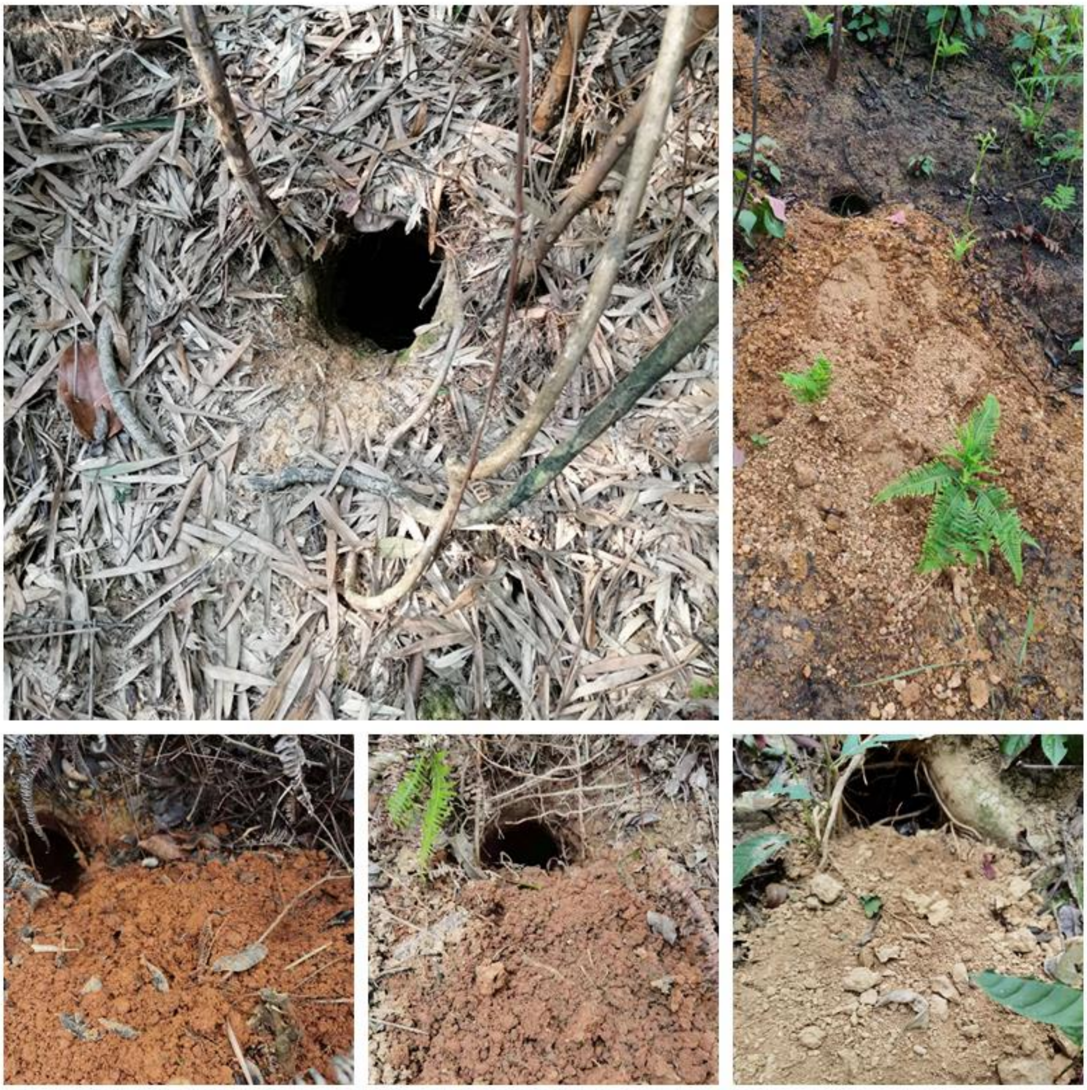

Table S1 Changes of soil properties

Soil property	Sample location	Heping region		Tianjingshan region		Wuqinzhang region	
		Mean (SD)	*P* value	Mean (SD)	*P* value	Mean (SD)	*P* value
Water content	Burrow	12.16 (±4.22)	<0.0001***	17.71 (±5.11)	0.0138 *	14 (±6.86)	0.021*
	Contrast	12.88 (±3.06)		21.21 (±4.63)		19.23 (±5.08)	
Sand content	Burrow	28.92 (±7.95)	<0.0001***	21.75 (±3.21)	0.237	22.4 (±5.75)	0.07
	Contrast	33.19 (±7.99)		20.94 (±3.59)		24.92 (±5.94)	
Unit weight	Burrow	1.01 (±0.14)	0.371	1.12 (±0.17)	0.889	1.09 (±0.18)	0.003**
	Contrast	0.99 (±0.17)		1.07 (±0.17)		0.90 (±0.12)	
pH	Burrow	3.97 (±0.19)	<0.0001***	3.97 (±0.16)	0.0398 *	4.77 (±0.49)	0.946
	Contrast	3.81 (±0.19)		3.84 (±0.12)		4.68 (±0.63)	
P content	Burrow	259.84 (±93.13)	<0.0001***	205.67 (±76.81)	<0.0001***	109.66 (±50.17)	0.008**
	Contrast	280.61 (±100.02)		231.39 (±72.65)		146.03 (±44.94)	
C content	Burrow	18.57 (±7.05)	0.0102 *	21.49 (±10.23)	0.21	9.14 (±4.13)	<0.0001***
	Contrast	29.94 (±8.44)		38.34 (±12.95)		34.67 (±7.93)	
Organic content	Burrow	32.02 (±12.15)	<0.0001***	37.05 (±17.63)	<0.0001***	15.75 (±7.12)	<0.0001***
	Contrast	51.62 (±14.54)		66.1 (±22.33)		59.78 (±13.68)	
Ammoniacal nitrogen	Burrow	0.19 (±0.13)	<0.0001***	0.21 (±0.13)	0.102	0.49 (±0.26)	0.001**
	Contrast	0.17 (±0.11)		0.29 (±0.15)		1.06 (±0.45)	
Nitrate nitrogen	Burrow	2.65 (±2.21)	<0.0001***	5.18 (±3.22)	<0.0001***	3.55(±2.6)	0.449
	Contrast	2.81 (±2.48)		5.45 (±2.89)		3.41 (±3.08)	
Microbial P content	Burrow	7.37 (±12.91)	0.0977	3.91 (±6.32)	<0.0001***	7.63 (±8.91)	0.094
	Contrast	11.79 (±6.32)		4.74 (±2.99)		8.72 (±5.04)	
Microbial C content	Burrow	28.98 (±39.61)	0.238	13.24 (±32.64)	0.288	37.74 (±69.88)	0.204
	Contrast	29.46 (±18.46)		8.18 (±3.25)		29.81 (±27.32)	
Microbial N content	Burrow	0.37 (±0.63)	0.0107 *	0.23 (±0.68)	0.068	0.37 (±0.88)	0.185
	Contrast	0.44 (±0.28)		0.13 (±0.06)		0.21 (±0.11)	

Note: Significant codes: 0.0001 '***', 0.001 '**', 0.01 '*', 0.05 '.', 0.1 ' ', 1
